# Flavivirus Infection and Regulation of Host Immune and Tissue Homeostasis in Insects

**DOI:** 10.3389/fimmu.2020.618801

**Published:** 2020-11-30

**Authors:** Sneh Harsh, Ioannis Eleftherianos

**Affiliations:** ^1^ Infection and Innate Immunity Lab, Department of Biological Sciences, Institute for Biomedical Sciences, The George Washington University, Washington, DC, United States; ^2^ Department of Biochemistry and Molecular Pharmacology, New York University School of Medicine, New York, NY, United States

**Keywords:** flavivirus, mosquito, *Drosophila*, immunity, homeostasis, pathophysiology

## Background

Flaviviruses are enveloped single-stranded RNA viruses and major human pathogens. They are responsible for causing outbreaks and therefore they represent a serious health issue worldwide (1). Because of the clinical significance of flaviviruses and the severity of epidemics they cause globally, developing efficient vaccines and drugs is critical for the success of disease control measures. Importantly, many flaviviruses, including Dengue virus (DENV), Japanese Encephalitis virus (JEV), West Nile virus (WNV), Yellow Fever virus (YFV), and Zika virus (ZIKV), are vectored though arthropods (arboviruses), mainly mosquitoes and ticks (2). Although most previous efforts have primarily focused on advancing the design of therapeutic strategies for alleviating the disease symptoms caused by flaviviruses, it is equally significant to also be able to interpret the molecular nature of the interactions that take place between flaviviruses and the insect vector and determine whether these interactions affect pathophysiological processes during infection and transmission.

Interactions between mosquito vectors and flaviviruses have been studied in several occasions (3). For instance, ZIKV is mainly transmitted by *Aedes aegypti* mosquitoes and recent studies have begun to examine vector-virus relationships and transmission dynamics of this virus pathogen (4). *Ae. aegypti* mosquitoes infected with ZIKV activate the RNA interference (RNAi) mechanism by upregulating several virus-produced short interfering RNAs (siRNAs), piwi-interacting RNAs and microRNAs. Many of the latter are also regulated by DENV and WNVs, but not by the alphavirus Chikungunya, indicating conservation in the mosquito response to flavivirus infection (5). Flavivirus infection in mosquito vectors activates innate immune signaling, which promotes the induction of antiviral response through the production of effector molecules (6). Activation of JAK/STAT together with Toll signaling elevates the *Ae. aegypti* resistance to ZIKV infection, silencing the Toll pathway adaptor MyD88 increases DENV infection in the *Ae. aegypti* midgut, and DENV infection in this mosquito vector decreases the signaling activity of immune deficiency (Imd) pathway (7–9). Interestingly, the secreted protein Vago limits WNV replication in *Culex* mosquito cells through induction of JAK/STAT signaling (10). Studies in mosquitoes are expected to contribute toward developing efficient procedures for preventing the spread of flaviviruses.

Results from research with insect vectors further stress the need for insect immunologists studying antiviral immunity to draw more attention to the outcome of flavivirus infection in insects rather than focusing exclusively on the replication efficacy of the virus. Therefore, this opinion article aims at highlighting recent studies that have started to dissect the interaction between antiviral immune mechanisms and flavivirus tropism as well as host tissue homeostasis and pathophysiological defects in mosquitoes and the model insect *Drosophila*. Such information is critical because it has the potential to lead to the development of novel therapeutics that will be directed against the ability of flaviviruses to multiply in certain insect organs. This will be valuable knowledge for understanding and potentially predicting the severity and extent of flavivirus infection and the efficiency of transmission.

## Flavivirus Interaction with Mosquito Immune and Metabolic Organs

The insect fat body is a diffused organ that functions similarly to the mammalian liver and is responsible for metabolism and storage of nutrients as well as the production and secretion of antimicrobial peptides and other immune factors (11). Flaviviruses present in the mosquito hemocoel (the insect body cavity) replicate in the abdominal and thoracic fat body before they disseminate to the salivary glands and other insect tissues (12). For instance, WNV replicates primarily in the fat body of *Culex pipiens quinquefasciatus* (13), but DENV replication has not been associated with this tissue in *Aedes albopictus* and *Ae. aegypti* mosquitoes (14, 15). However, more recent findings indicate that DENV replication in the fat body cells of *A. albopictus* alters the expression of Actin and alpha Tubulin (16), and downregulates the transcription of Toll pathway related genes in *Ae. aegypti* (8). In *Ae. aegypti*, YFV replicates in the fat body and other organs and feeding mosquitoes with a DENV-infected blood meal leads to activation of autophagy in the this tissue and the midgut and increases the expression of genes related to apoptosis such as the effector caspase *Casps7* (17–19). Notably, activation of JAK/STAT signaling specifically in the fat body of *Ae. aegypti* restrains DENV efficiently but fails to restrict ZIKV infection, and priming of these mosquitoes with inactive DENV induces the activation of Notch signaling upon infection with active DENV and reduces viral propagation in the midgut and carcass (20, 21). Flaviviruses transmitted by mosquitoes are taken up through a blood meal and migrate to the mosquito gut before they move to the salivary glands in order to be passed on to another vertebrate host and maintain their lifecycle (22). Main barriers to systemic infection include the midgut infection barrier, midgut escape barrier, salivary gland infection barrier, and salivary gland escape barrier (23). In the gut, flavivirus infection triggers the induction of antiviral pathways through interaction with receptors in the midgut epithelial cells (24). Interfering with the expression of certain RNAi signaling components in *Ae. aegypti* adult mosquitoes reduces DENV titers in the midgut following oral infection (25). Although Toll signaling plays a crucial role in the anti-DENV response in the midgut of *Ae. aegypti* (26, 27), the involvement of this pathway in the mosquito immune response against WNV and YFV is not fully determined yet (28). Also, Imd signaling activity participates in the induction of anti-DENV immune functions in the mosquito gut, because inhibition of this pathway leads to higher DENV load in *Ae. aegypti* midgut (29). Similar role has further been demonstrated for two DENV restriction factors, which are regulated by the JAK/STAT pathway and their expression lowers viral titers in the *Ae. aegypti* midgut (30). Apoptosis events have been found to occur in the midgut of *C. pipiens* and *Ae. aegypti* refractory strains during infection with WNV or DENV, respectively, indicating a potential role in restricting virus propagation by these mosquito vectors (31, 32). Strikingly, the presence of gut microbiota in *Ae. aegypti* can have a direct or indirect influence on DENV infection and spread either by enhancing the mosquito innate immune response or suppressing virus replication through the secretion of unknown molecules [(24, 33) and references therein]. DENV infection in *Ae. aegypti* can be affected by the midgut-inhabiting bacterium *Serratia odorifera* or the fungus *Talaromyces*, which both increase mosquito sensitivity to this virus. This is achieved through the production of a bacterial polypeptide that interacts with the virus or the modification of trypsin enzyme activity by the fungus. Both microbially mediated effects lead to considerable changes in mosquito physiology (34, 35). Interestingly, variation in *Wolbachia*-mediated DENV blocking in *Ae. aegypti* has been previously attributed to the production of nitric oxide or other free radicals (36). Also, serum ion can be utilized by the *Ae. aegypti* iron metabolism pathway to strengthen reactive oxygen species activity in the gut epithelium in order to oppose DENV infection (37). In addition, expression of the redox-sensing gene *nuclear factor erythroid-derived factor 2* (*Nrf2*) limits ZIKV infection by maintaining midgut homeostasis through modulation of reactive oxygen species in the midgut as well as microbiota growth and stem cell proliferation (38).

The association between flavivirus infection and lipid droplet regulation in mammalian cells has been previously reported (39–41). For example, infection of BHK-21, HepG2, and C6/36 cells by DENV increases markedly the number of lipid droplets per cell. This interaction possibly occurs between lipid droplets and various conserved residues in the core protein of the virus and probably indicates a link between viral replication and modulation of lipid metabolism (42). Also, during DENV infection of the hepatocyte derived cellular carcinoma cell line Huh7, HMG-CoA reductase activity increases, leading to higher cholesterol levels in the endoplasmic reticulum necessary for virus replication complex formation (43). In a similar fashion, infection of the *Ae. aegypti* cell line Aag2 with DENV increases lipid droplet accumulation (44). This phenotype in the DENV infected cells is associated with substantial upregulation of transcript levels of genes encoding factors related to lipid droplet biogenesis and lipid storage. These effects are connected with changes in immune signaling regulation, given that ectopic activation of Toll or Imd pathways further result in higher numbers of lipid droplets in the midgut.

## Lessons From the *Drosophila*-Flavivirus Model

**Figure 1:**
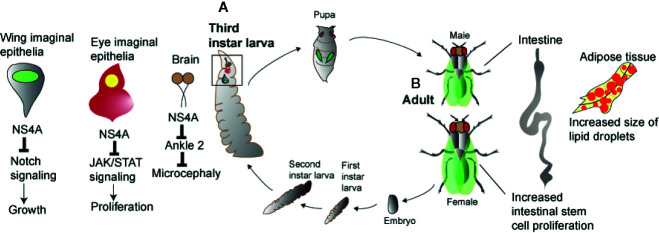
Zika virus and host pathologies in *Drosophila*. Zika virus results in perturbed homeostasis of different organs in *Drosophila* larva and adult. **(A)** Overexpression of Zika virus NS4A results in microcephaly in brain and restricted growth of the wing and eye imaginal epithelia. In the wing and eye imaginal epithelia, Zika virus NS4A interacts with Notch and JAK/STAT signaling pathways while in case of the brain, Zika virus NS4A targets ANKLE2, a highly conserved mitotic regulator. **(B)** Infection of Zika virus in *Drosophila* adult flies results in perturbed intestinal and adipose tissue homeostasis marked by increased intestinal stem cell proliferation and increased size of the lipid droplets.

Due to ZIKV outbreaks in several countries over the past five years, recent research has used the *Drosophila* model for leveraging the powerful genetic and genomic tools in the fly in order to understand flavivirus pathogenesis (**Figure 1**). ZIKV possesses a positive-sense single-stranded RNA genome encoding three structural (capsid, pre-membrane, envelope) and seven non-structural proteins (NS1, NS2A, NS2B, NS3, NS4A, NS4B, and NS5) (45). Expression of the ZIKV non-structural protein NS4A in the brain of *Drosophila* larvae induces apoptosis and leads to microcephaly, while expression of human *Ankyrin Repeat And LEM Domain Containing 2* (*ANKLE2*) gene, which is involved in brain development and has been previously implicated in hereditary microcephaly, in flies overexpressing ZIKV NS4A abolishes these defects. These results provide proof that ZIKV NS4A interacts physically with the ANKLE2 protein and causes microcephaly in an ANKLE2-dependent manner (46) (**Figure 1A**). More recently, these findings were extended by showing that mutations in *ANKLE2* gene perturbs an asymmetric cell division pathway in *Drosophila* neuroblasts and causes neurological disease and microcephaly, whereas overexpression of ZIKV NS4A in neuroblasts produces a similar phenotype observed in *Ankle2* mutants (47).

Also, it has been shown that ZIKV infection induces antiviral autophagy in the brain of adult *Drosophila* and this process depends on the activity of the Imd transcription factor Relish (48). The fly ortholog of the mammalian polyubiquitin-binding scaffold protein p62, the autophagy cargo receptor Ref(2)P, is also directed against ZIKV in the brain and protection against this pathogen is not dependent on RNAi signaling activity (48). Interestingly, ZIKV also replicates in the fat body, crop and gut of the adult fly and this tissue tropism disrupts gut and fat body lipid droplet homeostasis (49) (**Figure 1B**). This tissue-specific phenotype is further intensified in loss-of-function flies mutant for *Dicer-2*, the RNase of the RNAi pathway and is accompanied by reduced insulin signaling activity that leads to increased ZIKV replication and fly sensitivity to the infection (49). A genetic screen using naturally derived *Drosophila* lines revealed the insulin-like receptor InR as essential for fly survival to arbovirus infection (50). Insulin signaling was further found to suppress RNAi activity, but priming with mammalian insulin enhances the immune response to control ZIKV and DENV infection through induction of genes regulated *via* the JAK/STAT pathway.

Recently, it was demonstrated that ZIKV infection in *Drosophila* adult flies upregulates several gene targets that act as negative regulators of the JAK/STAT pathway and expression of certain ZIKV structural and non-structural proteins in different tissues of transgenic flies results in restricted eye growth, which is due to reduced rate of proliferation in eye imaginal epithelia (45). In particular, overexpression of ZIKV *NS4A*, a dominant negative form of *domeless*, and co-expression of dominant negative form of *domeless* and *NS4A* driven under an eye-specific promoter induces restricted eye phenotype in a JAK/STAT dependent manner. Of note, overexpression of ZIKV *NS4A* in the wing reduces the size of the pouch domain, an effect that is associated with decreased Notch signaling. This information points toward a relationship between ZIKV gene expression, JAK/STAT and Notch signaling activity which is necessary for *Drosophila* growth and development, and induction of pathological defects in the fly (**Figure 1A**). In a similar manner, expression of the DENV NS3 protein (which promotes virus replication) in *Drosophila* transgenic flies reduces their survival response to bacterial infection, but not to abiotic stress, indicating a link between DENV NS3 activity and antimicrobial immune capacity (51). Finally, a genome-wide RNAi screen in *Drosophila* cells has identified a large number of genes encoding cellular factors, many of which are able to restrict WNV infection. Intriguingly, all these genes are conserved in mosquitoes and the majority have human orthologs. Furthermore, a subset of those genes (e.g., dRUVBL1 and dXPO1) reduces flavivirus infection in adult flies demonstrating the power of *Drosophila* for the discovery of novel host molecules with anti-flavivirus activity (52).

## Conclusions and Perspectives

Flaviviruses have recently expanded globally by causing severe health impacts. Animal models are critical for understanding the molecular and physiological basis of host antiviral response and flavivirus pathogenesis. Because host innate immune responses are evolutionary conserved across many phyla, investigating the effect of flavivirus infection on the immune signaling and function of animal models is particularly informative because it can lead to the identification of anti-flavivirus immune processes in humans. Also, elucidating the scale of interactions between the host innate immune system and flaviviruses can lead to tissue-specific pathological deficits that modulate host functional changes associated with the disease. Probing the exact nature of interactions that take place during transmission of flaviviruses by mosquitoes and ticks and exploring the impact of the pathogens on tissue homeostasis during this process is considered a future research priority. Due to the close taxonomic relationship between mosquitoes and the common fruit fly (they are both members of the order Diptera), the use of *Drosophila* offers many advantages for studying these insect-borne viruses. Recent studies in *Drosophila* adult flies and larvae have been pivotal for the identification of fundamental mechanisms in insects that participate in the control of flaviviruses in the mosquito vector. If *Drosophila* factors interacting with flavivirus proteins are identified and characterized functionally, such findings could be extrapolated to mosquitoes after verification in the natural host (53). This approach could in turn put us in a better position to control the spread of flaviviruses in the mosquito vector, and thus enable us to prevent flavivirus dissemination to the human population.

## Author Contributions

SH wrote the original draft of the manuscript and IE revised and edited the paper. All authors contributed to the article and approved the submitted version.

## Conflict of Interest

The authors declare that the research was conducted in the absence of any commercial or financial relationships that could be construed as a potential conflict of interest.
